# Supplementary material for: Proteomic reprogramming of ileal epithelial cells during homologous superimposed intestinal trematode infection reveals coordinated restoration of intestinal homeostasis
Source: PLoS Negl Trop Dis. 2026 Aug 24;20(8):e0014294. doi: 10.1371/journal.pntd.0014294 (PMC13521460; doi:10.1371/journal.pntd.0014294)
Supplement: Supplementary file 1 [file pntd.0014294.s001.pdf]

```
#  C  = Control

#  I  = SuperInfected

#  SI = Super SuperInfected


library(glmnet)

library(car)

library(caret)

library(openxlsx)

library(NMF)


# Caso 3: Control vs SuperInfected

#

###########################################


datos<-read.csv2("datos.csv",  stringsAsFactors=FALSE, fileEncoding="latin1")

names(datos)

attach(datos)



Protein<-as.vector(datos[,1])


datos1<-data.frame(apply(datos[,-1],2,log2))


names(datos1)


#############################################

# Caso 1: Control vs SuperInfected vs Super SuperInfected

#############################################


#VEamos si hay diferencias para FR en los 4 tiempos
```

```
# Lasso: Diferencias entre los cuatro grupos

library(glmnet)

library(caret)

datos3<-data.frame(t(datos1[,c(1:4,9:12)]))

names(datos3)


#Lasso binomial :

#################################

##Comparaciones:


#1) Control vs SuperInfected


tipotrat <- factor(c(rep(0,4),rep(1,4)),levels=0:1,labels=c("Control","SuperInfected"))


normalizar<-t(scale(t(datos1[,c(1:4,9:12)])))


annotation = data.frame(type = tipotrat)

pdf(file="../LATEX/Figures/ControlvsSuperInfected/heatmap_normalizado.pdf",width =8,
height = 6,onefile= FALSE)

aheatmap(normalizar, scale="row", color=c("green",
"darkgreen","black","darkred","red"),annCol = annotation,labRow = NA,fontsize = 7,cexRow
= 0.7,annColors='Set1')

dev.off()

set.seed(123)

fit_lasso<-glmnet(as.matrix(datos3), tipotrat, nlambda=100, alpha=1, family="binomial")

plot(fit_lasso, label=TRUE)


cv <- cv.glmnet(as.matrix(datos3), tipotrat, alpha=1, nfolds=12, family="binomial")

plot(cv)

#Valor lambda con el que se consigue el mínimo test-error

l1<-cv$lambda.min  #0.03816708
```

```
lambdas.min<-replicate(100, cv.glmnet(as.matrix(datos3), tipotrat, alpha=1, nfolds=10, family="binomial")$lambda.min)

plot(density(lambdas.min))

median(lambdas.min)

l <- median(lambdas.min)   #0.03816708
l3<-cv$lambda.1se # 0.4491547
#Coeficientes del modelo
res <- as.matrix(predict(fit_lasso, newx=as.matrix(datos3),s=l, type="coefficients"))
res1 <- as.matrix(predict(fit_lasso, newx=as.matrix(datos3),s=l3, type="coefficients"))

coeficientes_lasso<-res[res!=0][-1]
coeficientes_lasso1<-res1[res1!=0][-1] #No tenemos coeficientes

#Filas seleccionadas(haciendo [-1] quitamos la interceptación
proteinas_lasso<-(which(abs(res)>0)-1)[-1]
# 594  618  738  867 1139 1394

proteinas_lasso_1se<-(which(abs(res1)>0)-1)[-1]
# 738

nom_proteinas_lasso<-Protein[proteinas_lasso]
Resultados_lasso<-data.frame(Fila=proteinas_lasso,Proteína=nom_proteinas_lasso,Coeficiente=coeficientes_lasso)

write.xlsx(Resultados_lasso, "../Resultados/ControlvsSuperInfected/Proteinas_lasso.xls",overwrite = TRUE)
```

```
#vamos a representar las proteínas identificadas con Lasso

pdf(file="../LATEX/Figures/ControlvsSuperInfected/heatmap_lasso.pdf",width = 6, height = 6,onefile= FALSE)

aheatmap(t(scale(datos3[,proteinas_lasso])), scale="row", color=c("green", "darkgreen","black","darkred","red"),annCol = annotation,labRow=nom_proteinas_lasso,fontsize = 7,cexRow = 0.7,annColors='Set1')

dev.off()

#################################
#VAmos a hacer Elastic Net
##############################

fit_net_bin<-glmnet(as.matrix(datos3), tipotrat, nlambda=100, alpha=1, family="binomial")
prueba<-glmnet(x = as.matrix(datos3), y=tipotrat,nlambda=100, alpha=0, family="binomial")   #[4.93,492.9]
prueba<-glmnet(x = as.matrix(datos3), y=tipotrat, nlambda=100, alpha=1, family="binomial")   #[0.00493, 0.4929]
#Para la secuencia de lambda se espacian más los valores de elevados de lambda
red <- expand.grid(.alpha = seq(0.1,1, 0.1), .lambda = exp(seq(log(0.00493),log(492.9), length=200)))

Control <- trainControl(verboseIter =TRUE,method="cv")
netFit <- train(x = as.matrix(datos3), y=tipotrat,method = "glmnet", tuneGrid = red, trControl = Control, family="binomial")

## alpha = 0.1, lambda = 2.27
plot(netFit)
attributes(netFit)
netFit$bestTune
```

```
a<-netFit$bestTune[,1]

l<-netFit$bestTune[,2]


datos5<-datos3


#VAmos a modificar el alpha y ponemos alpha=a

fit<-glmnet(as.matrix(datos5), tipotrat,alpha=a, family="binomial")  #alpha 1 = Lasso,
alpha 0=Ridge

plot(fit, label=TRUE)


res <- as.matrix(predict(fit, newx=as.matrix(datos5),s=l, type="coefficients"))


coeficientes_elasticNet<-res[res!=0][-1]


proteinas_elasticNet<-(which(abs(res)>0)-1)[-1]

nombre_proteina_elasticNet<-Protein[proteinas_elasticNet]


#4   9  10  19  24  25  32  34  35  42  58  59  62  67  73  88  89  92  94 101  106  108
110

# 114  117  126  127  132  153  155  161  162  183  185  186  188  192  196  212  284  297
322  328  329  346  356

# 358  359  360  375  392  403  422  430  454  463  464  473  480  487  489  492  508  512
529  555  565  581  591

# 594  618  620  635  636  638  639  654  666  693  702  722  727  735  738  784  787  793
795  807  842  846  857

# 859  860  866  867  869  881  893  897  925  949  987 1017 1020 1039 1048 1092 1105
1110 1139 1140 1145 1168 1171

# 1174 1190 1192 1215 1231 1236 1250 1259 1261 1289 1297 1318 1320 1338 1369 1384
1391 1394 1438 1459 1470 1477 1499

# 1501 1507 1567 1570 1614 1628 1689 1693 1716 1725 1738 1766


Resultados_elasticnet<-
data.frame(Variable=proteinas_elasticNet,Proteína=nombre_proteina_elasticNet,Coef=c
oeficientes_elasticNet)
```

```
write.xlsx(Resultados_elasticnet,
"../Resultados/ControlvsSuperInfected/Resultados_elasticnet.xls",overwrite = TRUE)


pdf(file="../LATEX/Figures/ControlvsSuperInfected/heatmap_elasticnet.pdf",width = 8,
height = 6,onefile= FALSE)

aheatmap(t(scale(datos3[,proteinas_elasticNet])), scale="row", color=c("green",
"darkgreen","black","darkred","red"),annCol = annotation,fontsize = 7,cexRow =
0.7,labRow=nombre_proteina_elasticNet,annColors='Set1')

dev.off()



#################################################

#

#plsda con el paquete mixOmics

#

#################################################

library(mixOmics)


datos3<-data.frame(t(datos1[,c(1:4,9:12)]))


#PCA (normal)

pca<- pca(datos3, ncomp = 3, scale = TRUE, center = TRUE)



pdf(file="../LATEX/Figures/ControlvsSuperInfected/pca.pdf",width = 8, height = 6,onefile=
FALSE)

plotIndiv(pca, group = tipotrat,col.per.group=c("red","blue"),

    title = 'PCA , comp 1 - 2',legend = TRUE)


dev.off()
```

```r
#PLS-DA :
##################


#PLS-DA classic
plsda <- plsda(datos3, tipotrat, ncomp = 2)



pdf(file="../LATEX/Figures/ControlvsSuperInfected/plsda.pdf",width = 8, height = 6,onefile= FALSE)
plotIndiv(plsda,
    group = tipotrat, ellipse = TRUE, col.per.group=c("red","blue"),
    legend = TRUE,
    title = 'Classical PLS-DA')

dev.off()



# R^2X: La primera componente explica un 41.6% de la varianza de las variables. La 2ª componente explica un 14.3% de la varianza de las variables que nos queda por explicar.

plsda$prop_expl_var$X

# R^2Y:La primera componente explica toda la varianza de la respuesta Y. La segunda componente es la varianza residual.Esto significa que la separación de los grupos ya se determina prácticamente toda con la primera componente y la segunda sólo aporta información marginal.

plsda$prop_expl_var$Y

##Validez y capacidad predictiva
perf.plsda <- perf(plsda,
        validation = "loo",
        progressBar = TRUE)
```

str(perf.plsda)

#Q^2 values: este valor para un n=4 no se puede estimar de forma fiable con validación cruzada por lo que vamos a dar el error de clasificación como medida aproximada del rendimiento del modelo. Esto nos dice que nos clasifica 1 muestra incorrectamente de las 8 que tenemos (4 en cada grupo)

perf.plsda$error.rate$overall

max.dist centroids.dist mahalanobis.dist

#comp1 0.125 0.125 0.125

#comp2 0.125 0.125 0.125

##

#la función vip nos da la importancia de cada una de las variables explicativas en la proyección, lo que nos va a

#permitir determinar cuál de las variables explicativas son más importante para predecir la variable respuesta.

##

#Aplicamos la función VIP-score, que calcula la influencia en la respuesta de cada

#predictor del modelo:

resultado.vip <- vip(plsda)

head(resultado.vip)

#nos quedamos sólo con los resultados que muestra cuando cogemos tres componentes y

#las ordenamos

vip.comp2<- resultado.vip[,1]

vip.ordenadas2 <- sort(vip.comp2)

#???Proteínas con un vip mayor a 1

proteinas_mayor1<-unique(names(which(vip.ordenadas2>=1)))

proteinas_mayor1<-as.factor(proteinas_mayor1)

proteinas_mayor1_num<-as.numeric(substr(proteinas_mayor1,2,5))

```
proteinas_plsdamayor1<-Protein[proteinas_mayor1_num]

proteinas_vip1<-data.frame(proteinas_mayor1_num,proteinas_plsdamayor1)

colnames(proteinas_vip1)<-c("NumProteina","Proteina")

write.xlsx(proteinas_vip1,
"../Resultados/ControlvsSuperInfected/Resultados_vip_mayor1.xls",sheetName =
"Proteinas")


#Para un VIP>1.5 no tenemos ninguna proteina

proteinas_mayor15<-unique(names(which(vip.ordenadas2>=1.5)))

proteinas_mayor15<-as.factor(proteinas_mayor15)

proteinas_mayor15_num<-as.numeric(substr(proteinas_mayor15,2,5))

sort(proteinas_mayor15_num)

proteinas_plsdamayor15<-Protein[proteinas_mayor15_num]

resultados_vipmayor15<-
data.frame(Fila=proteinas_mayor15_num,Proteínas=proteinas_plsdamayor15)


write.xlsx(resultados_vipmayor15,
"../Resultados/ControlvsSuperInfected/Resultados_vip_mayor15.xls",sheetName =
"Proteinas vip15",overwrite =TRUE)


##########################################################################
#########

#### Vamos a hacer un heatmap sólo para las proteinas con un vip>15 ###########

##########################################################################
#########

#


pdf(file="../LATEX/Figures/ControlvsSuperInfected/heatmap_vip1.pdf",width = 8, height =
6,onefile= FALSE)


aheatmap(t(scale(datos3[,proteinas_mayor1_num])), scale="row", color=c("green",
"darkgreen","black","darkred","red"),annCol =
annotation,labRow=proteinas_plsdamayor1,fontsize = 7,cexRow = 0.7,annColors='Set1')

dev.off()
```

```
pdf(file="../LATEX/Figures/ControlvsSuperInfected/heatmap_vip15.pdf",width = 8, height = 6,onefile= FALSE)

aheatmap(t(scale(datos3[,proteinas_mayor15_num])), scale="row", color=c("green", "darkgreen","black","darkred","red"),annCol = annotation,labRow=proteinas_plsdamayor15,fontsize = 7,cexRow = 0.7,annColors='Set1')

dev.off()

###############   ANALISIS CON PAQUETE LIMMA ################################

library("limma")

#Hemos hecho expresión diferencial para un caso sencillo de CtrolvsTrat pero podemos hacer todas las comparaciones e interacciones

#que consideremos interesantes:

#Hacemos todas las posibles combinaciones

#Proteinas que se transfieren a distintas temperaturas:

design <- model.matrix(~ 0+tipotrat)

colnames(design) <- c("Control","SuperInfected")

fit <- lmFit(datos1[,c(1:4,9:12)], design)

fit <- eBayes(fit)

#Coef especifica el contraste del modelo lineal de interés

topTable(fit,coef=1,number=1809,genelist=Protein,adjust.method="BH")

topTable(fit,coef=2,number=1809,genelist=Protein,adjust.method="BH")

#En la siguiente salida lo que hace es como un anova, ve las diferencias de los 4 tiempos(niveles) por eso saca el test F(en

#las salidas anteriores muestras el test t pq lo hace dos a dos. Las proteínas seleccionadas son las misma y los fold-change son
```

#los mismos. El valor AveExpr es la media de todos los fold-changes de los diferentes tiempos).

toptable_todas<-topTable(fit,number=1809,genelist=Protein,adjust.method="BH")

write.xlsx(toptable_todas, "../Resultados/ControlvsSuperInfected/toptable_individuales.xls",overwrite =TRUE)

#Vamos a ver las proteínas haciendo todas las posibles comparaciones:

#contrast.matrix <- makeContrasts(H0-L0, levels=design)

contrast.matrix <- makeContrasts(Control-SuperInfected, levels=design)

fit3 <- contrasts.fit(fit, contrast.matrix)

fit3 <- eBayes(fit3)

topTable(fit3,coef=1,number=1809,genelist=Protein,adjust.method="BH")

top_comparaciones<-topTable(fit3,number=1809,genelist=Protein,adjust.method="BH")

write.xlsx(top_comparaciones, "../Resultados/ControlvsSuperInfected/top_comparaciones.xls",overwrite =TRUE)

results3 <- decideTests(fit3,method="global")

data_results3<-summary(results3)

tabla_diamVenn_comparaciones<-data.frame(Protein,results3)

write.xlsx(tabla_diamVenn_comparaciones, "../Resultados/ControlvsSuperInfected/tabla_diamVenn_comparaciones.xls",overwrite =TRUE)

write.xlsx(data_results3, "../Resultados/ControlvsSuperInfected/tabla_diagrama_Venn.xls",overwrite =TRUE)

pdf(file="../LATEX/Figures/ControlvsSuperInfected/volcano_diferencias.pdf",width =8, height = 6,onefile= FALSE)

```
par(mfrow=c(2,1))

volcanoplot(fit3,coef=1,highlight=10,names=Protein,main="InfectedvsSuperInfected",cex
=0.6)

vennDiagram(results3, cex=0.8,

        include=c("up", "down"),

        counts.col=c("red", "blue"),

        circle.col = c("red", "blue", "green3"))

dev.off()


par(mfrow=c(1,1))
```
