# Supplementary material for: Proteomic reprogramming of ileal epithelial cells during homologous superimposed intestinal trematode infection reveals coordinated restoration of intestinal homeostasis
Source: PLoS Negl Trop Dis. 2026 Aug 24;20(8):e0014294. doi: 10.1371/journal.pntd.0014294 (PMC13521460; doi:10.1371/journal.pntd.0014294)
Supplement: Supplementary file 2 [file pntd.0014294.s002.pdf]

```
#  C  = Control

#  I  = SuperInfected

#  SI = Super SuperInfected


library(glmnet)

library(car)

library(caret)

library(openxlsx)

library(NMF)


# Caso 4: Infected vs SuperInfected

#


datos<-read.csv2("datos.csv",  stringsAsFactors=FALSE, fileEncoding="latin1")

names(datos)

attach(datos)



Protein<-as.vector(datos[,1])


datos1<-data.frame(apply(datos[,-1],2,log2))


names(datos1)


##############################################

# Caso 1: Control vs SuperInfected vs Super SuperInfected

##############################################


#VEamos si hay diferencias para FR en los 4 tiempos
```

```
#Lasso: Diferencias entre los cuatro grupos

library(glmnet)

library(caret)

datos3<-data.frame(t(datos1[,5:12]))

names(datos3)


#Lasso binomial :

#################################

##Comparaciones:


#1) Control vs SuperInfected


tipotrat <- factor(c(rep(0,4),rep(1,4)),levels=0:1,labels=c("Infected","SuperInfected"))


normalizar<-t(scale(t(datos1[,5:12])))


annotation = data.frame(type = tipotrat)

pdf(file="../LATEX/Figures/InfectedvsSuperInfected/heatmap_normalizado.pdf",width =8,
height = 6,onefile= FALSE)

aheatmap(normalizar, scale="row", color=c("green",
"darkgreen","black","darkred","red"),annCol = annotation,labRow = NA,fontsize = 7,cexRow
= 0.7,annColors='Set1')

dev.off()

set.seed(123)

fit_lasso<-glmnet(as.matrix(datos3), tipotrat, nlambda=100, alpha=1, family="binomial")

plot(fit_lasso, label=TRUE)


cv <- cv.glmnet(as.matrix(datos3), tipotrat, alpha=1, nfolds=12, family="binomial")

plot(cv)

#Valor lambda con el que se consigue el mínimo test-error

l1<-cv$lambda.min  #0.4640523
```

```
lambdas.min<-replicate(100, cv.glmnet(as.matrix(datos3), tipotrat, alpha=1, nfolds=10,
family="binomial")$lambda.min)

plot(density(lambdas.min))

median(lambdas.min)

l <- median(lambdas.min)  #0.4640523
l3<-cv$lambda.1se #  0.4640523
#Coeficientes del modelo
res <- as.matrix(predict(fit_lasso, newx=as.matrix(datos3),s=l, type="coefficients"))
res1 <- as.matrix(predict(fit_lasso, newx=as.matrix(datos3),s=l3, type="coefficients"))

coeficientes_lasso<-res[res!=0][-1]

#No tenemos coeficientes

#################################
#VAmos a hacer Elastic Net
#############################

fit_net_bin<-glmnet(as.matrix(datos3), tipotrat, nlambda=100, alpha=1,
family="binomial")
prueba<-glmnet(x = as.matrix(datos3), y=tipotrat,nlambda=100, alpha=0,
family="binomial")  #[4.64,464.1]
prueba<-glmnet(x = as.matrix(datos3), y=tipotrat, nlambda=100, alpha=1,
family="binomial")  #[0.00464, 0.4641]
#Para la secuencia de lambda se espacian más los valores de elevados de lambda
red <- expand.grid(.alpha = seq(0.1,1, 0.1), .lambda = exp(seq(log(0.00464),log(464),
length=200)))
```

```
Control <- trainControl(verboseIter =TRUE,method="cv")

netFit <- train(x = as.matrix(datos3), y=tipotrat,method = "glmnet", tuneGrid = red,
trControl = Control, family="binomial")


## alpha = 0.1, lambda = 0.0592

plot(netFit)

attributes(netFit)

netFit$bestTune



a<-netFit$bestTune[,1]

l<-netFit$bestTune[,2]



datos5<-datos3


#VAmos a modificar el alpha y ponemos alpha=a

fit<-glmnet(as.matrix(datos5), tipotrat,alpha=a, family="binomial")  #alpha 1 = Lasso,
alpha 0=Ridge

plot(fit, label=TRUE)


res <- as.matrix(predict(fit, newx=as.matrix(datos5),s=l, type="coefficients"))


coeficientes_elasticNet<-res[res!=0][-1]


proteinas_elasticNet<-(which(abs(res)>0)-1)[-1]

nombre_proteina_elasticNet<-Protein[proteinas_elasticNet]


#10  30  34  40  52  59  63  72  73  87  94  105  119  121  140  149  150  152  156  157  158
162  170
```

```
# 171 177 184 191 226 242 244 246 252 263 281 291 292 296 305 314 316 326
330 334 341 351 353

# 364 386 397 400 401 403 412 424 426 427 438 444 446 447 448 459 464 476
484 487 506 507 512

# 514 516 540 542 548 550 559 565 566 567 568 574 576 607 608 614 624 633
638 645 654 657 662

# 670 677 694 702 709 719 721 734 738 739 747 763 768 771 778 782 785 789
804 807 808 811 816

# 822 825 826 827 828 839 845 848 860 882 883 888 900 902 907 912 921 926
930 939 941 944 946

# 953 955 964 968 993 998 1000 1013 1021 1025 1028 1031 1032 1045 1052 1054 1057
1058 1061 1065 1074 1075 1076

# 1082 1107 1117 1122 1130 1132 1139 1142 1144 1145 1153 1154 1161 1165 1182 1187
1189 1190 1192 1194 1201 1202 1205

# 1229 1253 1263 1272 1273 1288 1291 1293 1298 1309 1310 1312 1318 1334 1344 1356
1363 1364 1366 1374 1379 1383 1389

# 1395 1401 1414 1426 1427 1433 1435 1436 1440 1442 1446 1449 1453 1457 1470 1471
1476 1488 1492 1494 1498 1500 1501

# 1504 1507 1509 1513 1515 1530 1535 1539 1540 1541 1542 1553 1562 1568 1571 1602
1606 1611 1617 1619 1628 1637 1638

# 1642 1643 1648 1657 1666 1672 1674 1677 1679 1704 1708 1716 1733 1736 1744 1750
1753 1754 1756 1766 1767 1781 1785

# 1792



Resultados_elasticnet<-
data.frame(Variable=proteinas_elasticNet,Proteína=nombre_proteina_elasticNet,Coef=c
oeficientes_elasticNet)


write.xlsx(Resultados_elasticnet,
"../Resultados/InfectedvsSuperInfected/Resultados_elasticnet.xls",overwrite = TRUE)


pdf(file="../LATEX/Figures/InfectedvsSuperInfected/heatmap_elasticnet.pdf",width = 8,
height = 6,onefile= FALSE)

aheatmap(t(scale(datos3[,proteinas_elasticNet])), scale="row", color=c("green",
"darkgreen","black","darkred","red"),annCol = annotation,fontsize = 7,cexRow =
0.7,labRow=nombre_proteina_elasticNet,annColors='Set1')
```

```
dev.off()


#################################################

#

#Vamos a hacer plsda con el paquete mixOmics

#

#################################################

library(mixOmics)


datos3<-data.frame(t(datos1[,5:12]))


#Hacemos un PCA (normal)

pca<- pca(datos3, ncomp = 3, scale = TRUE, center = TRUE)




pdf(file="../LATEX/Figures/InfectedvsSuperInfected/pca.pdf",width = 8, height = 6,onefile=
FALSE)

plotIndiv(pca, group = tipotrat,col.per.group=c("red","blue"),

    title = 'PCA , comp 1 - 2',legend = TRUE)


dev.off()


#PLS-DA  :

###################


#PLS-DA classic

plsda <- plsda(datos3, tipotrat, ncomp = 2)


pdf(file="../LATEX/Figures/InfectedvsSuperInfected/plsda.pdf",width = 8, height =
6,onefile= FALSE)

plotIndiv(plsda,

    group = tipotrat, ellipse = TRUE, col.per.group=c("red","blue"),
```

```
        legend = TRUE,
        title = 'Classical PLS-DA')

dev.off()
```

R^2X: La primera componente explica un 19.8% de la varianza de las variables. La 2ª componente explica un 16.3% de la varianza de las variables que nos queda por explicar.

```
plsda$prop_expl_var$X
```

R^2Y:La primera componente explica toda la varianza de la respuesta Y. La segunda componente es la varianza residual.Esto significa que la separación de los grupos ya se determina prácticamente toda con la primera componente y la segunda sólo aporta información marginal.

```
plsda$prop_expl_var$Y
```

##Validez y capacidad predictiva

```
perf.plsda <- perf(plsda,
            validation = "loo",
            progressBar = TRUE)
str(perf.plsda)
```

#Q^2 values: este valor para un n=4 no se puede estimar de forma fiable con validación cruzada por lo que vamos a dar el error de clasificación como medida aproximada del rendimiento del modelo. En este caso el modelo no separa bien las muestras. Nos dice que 5 de 8 muestras se clasifican mal, luego el modelo en este caso casi es aleatorio, luego la clasificación que vemos de forma visual puede no ser real.

```
perf.plsda$error.rate$overall

#      max.dist   centroids.dist   mahalanobis.dist
#comp1   0.625       0.625          0.625
#comp2   0.500       0.625          0.500
```

```
##########################################################################
##########################################

#la función vip nos da la importancia de cada una de las variables explicativas en la proyección, lo que nos va a

#permitir determinar cuál de las variables explicativas son más importante para predecir la variable respuesta.

##########################################################################
##########################################

#Aplicamos la función VIP-score, que calcula la influencia en la respuesta de cada

#predictor del modelo:


resultado.vip <- vip(plsda)

head(resultado.vip)

#nos quedamos sólo con los resultados que muestra cuando cogemos tres componentes y

#las ordenamos

vip.comp2<- resultado.vip[,1]

vip.ordenadas2 <- sort(vip.comp2)

#???Proteínas con un vip mayor a 1

proteinas_mayor1<-unique(names(which(vip.ordenadas2>=1)))

proteinas_mayor1<-as.factor(proteinas_mayor1)

proteinas_mayor1_num<-as.numeric(substr(proteinas_mayor1,2,5))

proteinas_plsdamayor1<-Protein[proteinas_mayor1_num]

proteinas_vip1<-data.frame(proteinas_mayor1_num,proteinas_plsdamayor1)

colnames(proteinas_vip1)<-c("NumProteina","Proteina")

write.xlsx(proteinas_vip1, "../Resultados/InfectedvsSuperInfected/Resultados_vip_mayor1.xls",sheetName = "Proteinas")



#Para un VIP>1.5 no tenemos ninguna proteina

proteinas_mayor15<-unique(names(which(vip.ordenadas2>=1.5)))

proteinas_mayor15<-as.factor(proteinas_mayor15)

proteinas_mayor15_num<-as.numeric(substr(proteinas_mayor15,2,5))
```

```
sort(proteinas_mayor15_num)

proteinas_plsdamayor15<-Protein[proteinas_mayor15_num]

resultados_vipmayor15<-
data.frame(Fila=proteinas_mayor15_num,Proteínas=proteinas_plsdamayor15)


write.xlsx(resultados_vipmayor15,
"../Resultados/InfectedvsSuperInfected/Resultados_vip_mayor15.xls",sheetName =
"Proteinas vip15",overwrite =TRUE)


########################################################################
#########

#### Vamos a hacer un heatmap sólo para las proteinas con un vip>15 ###########

########################################################################
#########

#


pdf(file="../LATEX/Figures/InfectedvsSuperInfected/heatmap_vip1.pdf",width = 8, height =
6,onefile= FALSE)


aheatmap(t(scale(datos3[,proteinas_mayor1_num])), scale="row", color=c("green",
"darkgreen","black","darkred","red"),annCol =
annotation,labRow=proteinas_plsdamayor1,fontsize = 7,cexRow = 0.7,annColors='Set1')

dev.off()


pdf(file="../LATEX/Figures/InfectedvsSuperInfected/heatmap_vip15.pdf",width = 8, height
= 6,onefile= FALSE)


aheatmap(t(scale(datos3[,proteinas_mayor15_num])), scale="row", color=c("green",
"darkgreen","black","darkred","red"),annCol =
annotation,labRow=proteinas_plsdamayor15,fontsize = 7,cexRow = 0.7,annColors='Set1')

dev.off()


###############   ANALISIS CON PAQUETE LIMMA
###############################
```

```
library("limma")


#Hemos hecho exspresión diferencial para un caso sencillo de CtrolvsTrat pero podemos hacer todas las comparaciones e interacciones

#que consideremos interesantes:


#Hacemos todas las posibles combinaciones

#Proteinas que se transfieren a distintas temperaturas:


design <- model.matrix(~ 0+tipotrat)

colnames(design) <- c("Infected","SuperInfected")


fit <- lmFit(datos1[,5:12], design)

fit <- eBayes(fit)

#Coef especifica el contraste del modelo lineal de interés

topTable(fit,coef=1,number=1809,genelist=Protein,adjust.method="BH")

topTable(fit,coef=2,number=1809,genelist=Protein,adjust.method="BH")

#En la siguiente salida lo que hace es como un anova, ve las diferencias de los 4 tiempos(niveles) por eso saca el test F(en

#las salidas anteriores muestras el test t pq lo hace dos a dos. Las proteínas seleccionadas son las misma y los fold-change son

#los mismos. El valor AveExpr es la media de todos los fold-changes de los diferentes tiempos).


toptable_todas<-topTable(fit,number=1809,genelist=Protein,adjust.method="BH")

write.xlsx(toptable_todas, "../Resultados/InfectedvsSuperInfected/toptable_individuales.xls",overwrite =TRUE)


#Vamos a ver las proteínas haciendo todas las posibles comparaciones:

#contrast.matrix <- makeContrasts(H0-L0, levels=design)

contrast.matrix <- makeContrasts(Infected-SuperInfected, levels=design)

fit3 <- contrasts.fit(fit, contrast.matrix)

fit3 <- eBayes(fit3)
```

```
topTable(fit3,coef=1,number=1809,genelist=Protein,adjust.method="BH")

top_comparaciones<-topTable(fit3,number=1809,genelist=Protein,adjust.method="BH")
write.xlsx(top_comparaciones,
"../Resultados/InfectedvsSuperInfected/top_comparaciones.xls",overwrite =TRUE)

results3 <- decideTests(fit3,method="global")
data_results3<-summary(results3)

tabla_diamVenn_comparaciones<-data.frame(Protein,results3)
write.xlsx(tabla_diamVenn_comparaciones,
"../Resultados/InfectedvsSuperInfected/tabla_diamVenn_comparaciones.xls",overwrite
=TRUE)

write.xlsx(data_results3,
"../Resultados/InfectedvsSuperInfected/tabla_diagrama_Venn.xls",overwrite =TRUE)

pdf(file="../LATEX/Figures/InfectedvsSuperInfected/volcano_diferencias.pdf",width =8,
height = 6,onefile= FALSE)
par(mfrow=c(2,1))
volcanoplot(fit3,coef=1,highlight=10,names=Protein,main="InfectedvsSuperInfected",cex
=0.6)
vennDiagram(results3, cex=0.8,
        include=c("up", "down"),
        counts.col=c("red", "blue"),
        circle.col = c("red", "blue", "green3"))
dev.off()

par(mfrow=c(1,1))
```
